# Hospital Meetings, &c.

**Published:** 1899-07-15

**Authors:** 


### HOSPITAL MEETINGS, &C.

### HOSPITAL FOR EPILEPSY AND PARALYSIS.

The thirty-second annual meeting of the Hospital for Epilepsy and Paralysis and other Diseases of the Nervous System was held on Wednesday, 5th inst., the Earl of Hardwicke, president of the institution, in the chair.

The report, read by the secretary, Mr. Graham, stated the year had been one of great anxiety to the committee of management, owing to the fact, which they felt they could not too often or too emphatically impress upon their supporters, that in April next the lease of the premises so long occupied by the hospital would expire without hope of renewal. It was therefore essential to the future of the institution that they should acquire or build suitable premises at the earliest possible date, so that the work might go on with the least possible interruption. With the last annual report a statement was issued showing that nearly £2,500 was then in hand for the New Building Fund, but it was not until after the end of 1898—the year to which that report should properly be confined—that, in response to an appeal issued by their new president, the supporters of the hospital began to send contributions. Many old friends had, however, not yet been heard from, and the committee hoped that all such would at once come to their aid. On December 31st, 1898, the fund had reached a total of £3,294, and since then a bequest of £1,000 and further donations had brought it up to over £4,700. This sum is only about one fourth of what is required for an efficient and adequate structure, but the committee stated that under a will by which the fund had already benefited they hoped to receive a further sum of £3,000; while another "discretionary" bequest might produce £1,000 or £2,000 more. On the other hand, great disappointment had been caused by their learning that, owing to the insufficiency of the funds at the disposal of the testatrix, the £4,000 they had been led to expect under the will of the late Mrs. E. Armitage would not be forthcoming. Regret was also felt that no grant had been received from the Prince of Wales's Fund that year, but this was mitigated by the receipt of a gift of ten guineas from one of the visitors of the council. Mention was made of the fact that all patients are encouraged to contribute towards the expenses of the hospital, and that the principle was steadily gaining in favour. Since the end of 1898 the Earl of Hardwicke had been elected president in the room of the Earl of Chichester, whose resignation of the office held by him since 1881 had been accepted with much regret. The committee placed on record their sense of indebtedness to Lord Chichester for his valuable help during the past eighteen years, and also to Lord Hardwicke for consenting to be elected, and for the appeal he had circulated, bringing in many liberal donations. The number of in-patients under treatment during 1898 was two more than the average of recent years. The great increase in attendances of out-patients has been further augmented, reaching 12,562, as compared with 11,429 in 1897.

The Chairman, in moving the adoption of the report, said no better proof of the increasing interest in nervous disorders and recognition of the necessity for provision for their alleviation could be found than in the fact that the London County Council had arranged for 300 patients to be dealt with; but, being a public body, these cases could only be those of the obviously insane. As to the necessity for the continuance of the hospital, there could be no question, but if it were to be continued a very great effort must be made to get together the £8,000 still necessary.

Lord Farquhar seconded the motion, which was agreed to, and Canon Barker and General Lowry having spoken, the proceedings terminated.

### THE ALEXANDRA HOSPITAL FOR CHILDREN WITH HIP DISEASE.

The new Alexandra Hospital, which has been built for the reception of children with hip disease at Queen's Square, Bloomsbury, is rapidly approaching completion. It is a substantial building of unpretentious exterior, standing in a pleasant, quiet corner of the square. On the ground floor, on the right of the handsome entrance hall, are the board-room, office, waiting-room, side entrance, and surgeon's room, with lavatories and other conveniences. On the left is the lady superintendent's sitting-room, the nurses' dining-room, staircase, and lift for food, &c. On the first floor and on that immediately above are two wards, those in front having five large windows overlooking the square and being ventilated by shafts opening on the inside wall with fans above the windows opposite. They are heated by radiators. The smaller wards at the back overlook the garden, and are heated by square stoves in the centre. There is also a ward kitchen on each floor fitted with gas stoves and communicating with the kitchen in the basement by food lifts. On the third and fourth floors and in the attics are bedrooms for the use of the nursing and domestic staffs, linen-rooms, box-rooms, &c. From the third floor an iron balcony gives the only access to the isolation department. It consists of a couple of small wards, a nurses' room, kitchen, linen closet and disinfecting chamber, the last-named being separated from the rest of the floor by an additional fireproof door. The flooring throughout is of oak blocks laid on concrete, the halls and landings are paved with terrazzo, the steps are stone, the bannisters iron. The upper parts of the walls are distempered in pale green, and the lower painted with a dado of slightly deeper tone, corresponding with the woodwork. The effect is exceedingly pleasant. In the basement are the kitchen, pantries, servants' hall, and housekeeper's office. There is also a small laundry fitted with machinery worked by hand power only. There is ample cellarage, and in the boiler-room there is one boiler for heating the water for domestic purposes and a second for supplying heat to the radiators. A beautiful little mortuary is placed at the bottom of the garden, well screened from view by overhanging trees. It is ecclesiastical in appearance, and has an inner chamber which is all that can be desired for post-mortem examinations. Thr architects are Messrs. Hewitson, of Upper Brook Street. The cost of the new hospital is £20,000. Of that sum there only remains £500 to be subscribed. The Prince and Princess of Wales have promised to open it on July 20th.

